# Botanical Report

**Published:** 1812-01

**Authors:** 


### BOTANICAL REPORT.

THE last number of the Botanical Magazine may very well serve as a specimen of Mr. Sydenham Edwards's talents as a botanical draughtsman. This excellent artist appears to us to continue to improve; and we know of none that expresses with more truth and natural ease the habit or mode of growth of the plant; or that contrives better to display the parts of fructification, which can be seen withoutwithout dissection; and this is the more important, as the plan of the work does not admit the latter illustration. There are artists who finish higher; but such additional labor would be useless for a work like the present, which is sold at so low a price, that it could not possibly afford the increased expense that must have attended the engraving and coloring of drawings finished in a higher style; nor for the purpose of the botanist, or to assist in the knowledge of the species, would any advantage accrue from the greater labor bestowed. Ehret, in the last century, appears to us to have drawn plants far more naturally, and with less affectation, than any of his competitors; and Edwards, if we mistake not, for his style of botanical drawing, has taken him for his model.

This artist has been fortunate too, in having his drawings copied by excellent engravers; the late Mr. Sansum was minutely accurate, and faithfully copied the drawing before him—and his son, with all his father's accuracy, unites a more delicate hand, and finishes the tender blossoms with a softer touch.

As to coloring, we know of no colored work on natural history, that can stand the test of a comparison with the Botanical Magazine. Let any one compare the figures in this cheap performance, with the splendid and costly colored engravings that have, of late, issued from the Paris press, (having, at the same time, living specimens of the plants represented, in his hand), and we have no doubt of the general superiority of the former, as approaching nearest to nature. We believe Mr. Graves was the first person who, under the eye of the late Mr. Curtis, established a manufactory for coloring botanical engravings, of any account in this city; and he still continues to maintain his superiority, whilst a large portion of the best hands, in every department of natural history, have issued from his school.

This number contains:

PANCRATIUM *amboinense*. A rare plant from the collection of James Vere, esq. Mr. Ker has taken the opportunity of giving a new character to this genus; which, as it now stands, is not very definite; the cells of the germen being described as bearing many seeds, or few and definite. The seeds in the capsule vary from many to a solitary one; but, as this arises, or is supposed to arise, from abortion, it is of less consequence than the number of ovula in the germen. The present species having only two ovula in each cell, Mr. Brown, in his Prodromus, has remarked that it differs in this respect from all the rest of the genus, and also in the division of the corona, and approaches, as also in habit, to one of his New Holland genera, named Calostemma. Mr. Ker, however, could not observe any difference in the crown. We observe by the figure of this plant in the Paradisus Londinensis that the foliation is involute. We do not know how far this circumstance may be thought to confirm Mr. Brown's idea of a generic difference.

Allium *pallens*. The genus Allium contains a great number of species, and the proper designation of each is attended with much difficulty; notwithstanding the assistance given by Haller, who wrote a monograph upon it. This is not, however, according to Mr. Ker, the the *pallens* of that author, which belongs to flavum; nor of Brotero or Redouté. Mr. Ker always takes great pains to correct the synonymy, which is certainly of great importance, as nothing tends so much to confuse the knowledge of a plant as an assemblage of false synonyms. The French botanists are apt to be particularly careless in this respect.

Asthropodium *paniculatum*. This is the Anthericum Paniculatum of the Botanist's Repository—*milleflorum* of Redouté: is a native of New Holland, and considered by Mr. Brown as a distinct genus; but, though Mr. Ker has adopted this author's name, he has positively stated that it is in no respect distinct from Jussieu's genus Phalangium, which is usually united with Anthericum. Judging from the habit alone, we have always suspected it to be distinct from Anthericum; but, if Mr. Ker was convinced that it was not, he ought to have kept it with that genus.

Pœonia *humilis*. This is, we doubt not, distinct from P. *peregrina*, before figured in the Magazine; though the difference would have been more obvious, had the outline of a full-sized leaf been given, which the size of the plate would not admit of; we cannot, however, but repeat our regret that a double sized plate is not upon all occasions had recourse to when the true character of the plant cannot be given without it.

Justicia *bicolor*. This seems to be quite a new species, native of Luconia, one of the Philippine islands, whence the seeds were sent by Mr. William Kerr, who, we believe, is one of the gardeners sent into foreign parts by his majesty, to collect plants for the royal garden at Kew. It appears to be an acquisition to our stoves. The name seems to have been suggested by some similarity in the form of the flower to Viola *tricolor*.

Penstemon *pubescens* and *lævigata*. These two species are well brought together, as their distinction is not generally known, the latter having probably been seldom seen in our gardens of late years, the narrow-leaved variety of the former being usually taken for it. The character taken from the difference in the hairiness of the barren filament, and represented by an outline in both plates, seems by Dr. Sims's account to be liable to some exception, and a more obvious one is found in the nearly-naked panicle of the latter. These two figures are beautifully executed.

We have received another Number of the Botanist's Repository, containing

Prostanthera *lasianthos* of Labillardiere, a new acquisition from Van Diemen's island, raised in Lord Grenville's garden at Dropmore. By uniting the generic character with a specific description, the author has made a ridiculous jumble. And, though he quotes Labillardiere's New Holland Plants, he does not seem to have seen that work, or he could hardly have designated it by the name of his "Nova Hollandia, or a Description of New South Wales!" This remarkable plant belongs to Didynamia gymnospermia, bearing, according to the character, four berries, in the room of four naked seeds. These, Brown says, are not perfect berries. This last author describes twelve twelve other species, though Mr. Andrews supposes the genus contains a solitary one only.

Gompholobium *grandiflorum.* A very fine species from the conservatory of the Comptesse de Vandes.

Justicia *bicolor.* The same as mentioned above from the Botanical Magazine, said here, but erroneously, to be a native of Jamaica. The figure is far inferior to the other.

Lobelia *speculum.* A species nearly related to L. *unidentata,* but sufficiently distinct from that and every other, by the greater regularity of the limb of the corolla. In his specific character the author describes it as a dwarf shrub, but he afterwards says it is a delicate slender little annual: the last character we believe is the true one. We observed this little plant last summer at Mr. Colville's. Mr. Andrews must certainly mistake, when he asserts that the late Dr. Solander had an intention to separate it by the generic title of *Speculare.*

EPIDENDRUM *fragrans.* There is a figure of this plant in the 5th volume of the Botanical Magazine, though under a false name; and, either from an accidental variation, or from a supposed defect of a leaf, mistakenly represented with two leaves; both which circumstances are corrected by Dr. Sims, in the Index and Enumeration to the first twenty volumes of the Magazine. The price of the Botanist's Repository is raised to 6*s.* the number.

We are sorry to find that, in the great commercial prosperity of the island of Malta, the botanic garden there, which once promised better things, has been suffered to go entirely to decay, and is now made a public parade. We understand that in a private garden there the Plantain tree (the yellow-fruited variety) has born fruit last summer, equal in size and flavor to the product of the tropical climates.

